# Strategies for diagnosis of fetal right atrium dilation: based on fetal cardiac anatomy and hemodynamics

**DOI:** 10.1186/s12880-020-00477-0

**Published:** 2020-07-06

**Authors:** Yu Wang, Leisheng Zhao, Ying Zhang

**Affiliations:** Department of Ultrasound, Shengjing Hospital of China Medical University, NO.36 Sanhao Street, Heping District, Shenyang, 110004 Liaoning Province China

**Keywords:** Fetal echocardiography, Right atrium dilation, Hemodynamics

## Abstract

**Background:**

Fetal right atrium (RA) dilation is frequently detected in routine screenings while it remains a challenge to clarify the reasons. This study aimed to analyze the cardiac anatomy and hemodynamics of fetal RA dilation and the changes of hemodynamic indexes.

**Methods:**

In the retrospective study, 420 fetuses with RA dilation were included, which were classified into the physiological group (*n* = 202), volume overload group (*n* = 142), and the pressure overload group (*n* = 76). The ratio of right atrium to left atrium (RA/LA) were measured at four-chamber view. Peak velocity of tricuspid regurgitation (V_TR_) was recorded in each case, if existed.

**Results:**

The RA/LA ratio in the volume overload group is significantly higher than both the pressure overload group and the physiological group (both *P* = 0.000) throughout the pregnancy while no difference presents between the pressure overload group and the physiological group (*P* = 0.694 for 19–31 GW, and *P* = 0.974 for 32–36 GW, respectively). The V_TR_ in the pressure overload group (3.29 ± 0.58 m/s) is significantly higher than both the volume overload group (1.85 ± 0.45 m/s, *P* = 0.000) and the physiological group (0.88 ± 0.45 m/s, *P* = 0.000). The volume overload group shows a significantly higher V_TR_ than the physiological group (*P* = 0.000). In the volume overload group, the ductal contraction/closure shows a significantly higher V_TR_ than that in the pulmonary valve stenosis/atresia (3.98 ± 0.41 m/s vs. 3.03 ± 0.38 m/s, *P* = 0.000).

**Conclusions:**

A strategy proposed herein is useful to clarify the reasons for RA dilatation by systematically assessing fetal hemodynamics, which may facilitate the sonographers to make an accurate diagnosis of congenital heart disease.

## Background

Congenital heart disease (CHD), accounting for about 0.4–1.3% of all live births [1–4], is the most common congenital malformation leading to perinatal morbidity and mortality and is considered the leading cause of death in newborn with congenital anomalies [5–7]. Fetal echocardiography can undoubtedly make a comprehensive evaluation for CHDs [8]. However, the fetal cardiac echo is in needs of much experience and expertise for routine screening sonographers given its difficulty.

Right atrium (RA) dilation is frequently detected in routine screenings while it is difficult for sonographers to clarify the reasons of the enlarged chamber, either a normal physiological sign or a secondary event caused by intra-cardiac or extra-cardiac anomalies. In fact, many cardiac abnormalities have an enlarged RA as an associated sign which attracts attention of sonographers rather than the deficiency itself [9]. In the case of fetal pulmonary valve atresia with a well-developed pulmonary artery, the abnormal valve is not evident enough to be observed at first glance while an enlarged RA together with tricuspid regurgitation (TR) could often be identified during routine obstetric examinations. If the fetus is referred to a further examination by an echocardiography specialist, the correct diagnosis may be reached. Unfortunately, many scans just stop here with the cardiac lesions resulting in RA dilation not detected, especially in late pregnancy when RA dilatation is always considered as a normal physiological sign.

In the current study, we presented data from a 10-year cohort of cases with echocardiographic findings. We aimed to propose a strategy to clarify the reasons for RA dilatation by systematically evaluating fetal hemodynamics alterations in different conditions.

## Methods

### Study subjects

We retrospectively reviewed the image data obtained during routine obstetric examinations and fetal echocardiograms from the fetal screening center and echocardiography center in our hospital from Jan 2007 to Dec 2016. We searched our database in the diagnosis including RA dilation and found 464 fetuses of the review. Outcome data was obtained from hospital records (postnatal echocardiography, autopsy or operation) and phone-call follow up. In total, 420 fetuses with confirmed diagnosis were included in the current study. All the fetuses were singletons. Only data from the first echocardiogram were included in the current study in the case that the same patient had undergone several examinations. Fetal biparietal diameter and femur length were used to determine gestational age. RA dilatation between 19 and 31 gestational weeks was defined an estimated RA/LA > 1.1, 32 gestational weeks and above was defined > 1.2, established by Tan J, et al. [10].

This study was approved by the Ethics Committee of Shengjing Hospital of China Medical University. Written informed consent was obtained from the parents for publication of clinical details, clinical images, and videos.

### Fetal cardiac examination

All fetuses with normal or abnormal cardiac structures were examined using ultrasound systems (Voluson 730, E8, and E10, GE Healthcare, Kretztechnik, Zipf, Austria). At first, routine obstetric sonography was performed to detect extra-cardiac malformations. Then a detailed cardiac examination was performed by an experienced fetal echocardiographer. The visceral and cardiac position was determined as previously described [10]. Briefly, a long-axis plane of the fetus combined with the transverse planes at both the fetal abdominal level and thoracic level was used to ascertain whether both the stomach and heart were on the left side of the fetus. Four transverse views including the four-chamber view (4CV), the left and right outflow tract views, and the three-vessel trachea (3VT) view were scanned. In addition, three sagittal views including bi-caval view, aortic arch view, and the ductal arch view were also scanned. Color Doppler, pulsed Doppler, and/or high-definition flow imaging were performed when necessary. All image data was saved as video clips for later analysis. Diameters of RA and LA were measured in systole from the lateral walls of each atrium to the edge of septum secundum at a 4CV, established by Tan J, et al. [11]. The ratio of RA/LA in diameter was calculated. Peak velocity of tricuspid regurgitation (V_TR_) was recorded in each disease. All dimensions were measured three times, and the average value was used.

### Statistical analysis

Data were expressed as mean ± standard deviation. Differences between multiple means were compared by one-way ANOVA, using Tamhane’s T2 test or Bonferroni test when the variance was heterogeneous or homogeneous, respectively. All tests were considered significant when *P* < 0.05.

## Results

### Disease types

**Fig. 1:**
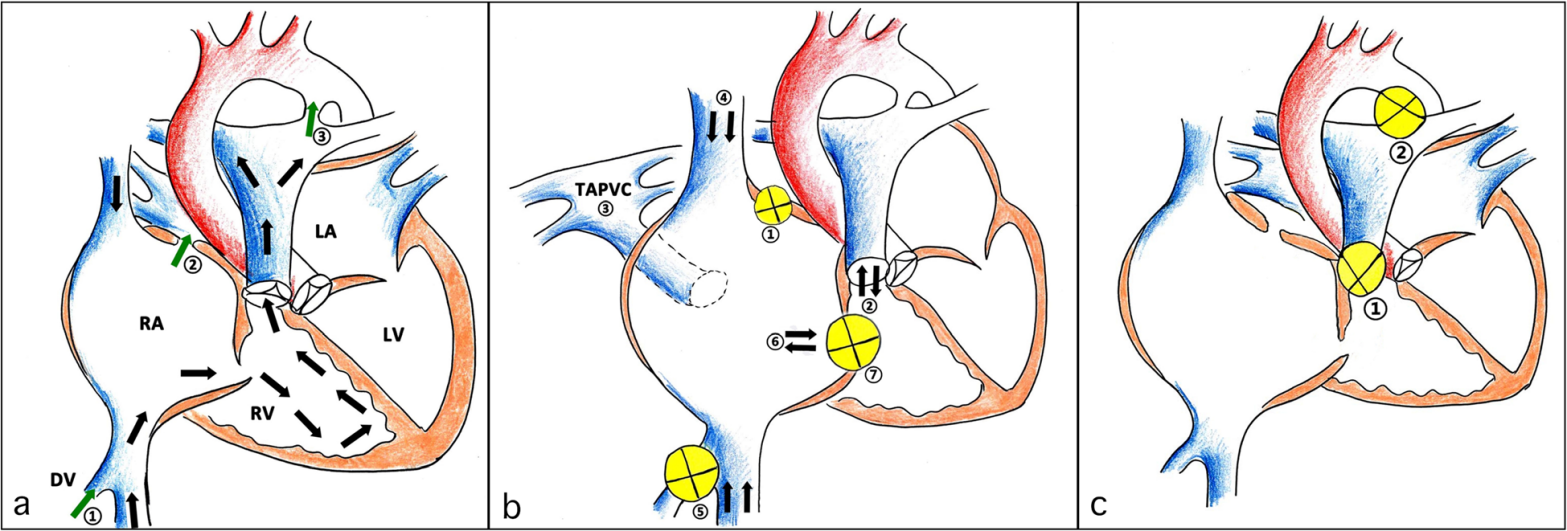
Illustrations showing fetal normal and abnormal hemodynamics. The normal fetal hemodynamics are illustrated in (**a**). The flow direction of right heart circulation is indicated by the black arrows. Blood flow direction at DV (①), FO (②), and the DA(③) are indicated by three green arrows. The hemodynamics of increasing RA volume are illustrated in (**b**), which contains several anomalies, such as restrictive FO (①), pulmonary regurgitation (②), TAPVC (③), increasing blood volume in SVC (④) and IVC (⑤), tricuspid dysplasia (⑥) and atresia (⑦), and so on. The hemodynamics of increasing right heart pressure are illustrated in (**c**). The associated cardiac anomalies are pulmonary stenosis/atresia (①) and contraction/closure of DA (②). DA: ductus arteriosus; DV: ductus venous; FO: foramen ovale; IVC: inferior vena cava; LA: left atrium; LV: left ventricle; RA: right atrium; RV: right ventricle; SVC: superior vena cava; TAPVC: total anomalous pulmonary venous connection

In total, 420 fetuses with RA dilation were included in the current study. RA dilatation may present in many cardiac malformations, or, in some cases, appear in normal fetuses in late pregnancy as a result of increasing blood volume in the right heart system. These fetuses with dilated RAs were then classified into three types: physiological RA dilatation (physiological group, *n* = 202), the increasing of RA volume (volume overload group, *n* = 142), and the increasing of right heart pressure (pressure overload group, *n* = 76). Hemodynamics in normal fetuses and in those with RA dilatation were illustrated in a sketch map (Fig. 1).

Among the reasons for RA dilation, physiological enlargement was undoubtedly the most common, especially in late pregnancy. For these 202 fetuses, 184 cases showed a normal RA/LA within the first 2 weeks of life by postnatal echocardiogram, 133 of which had an opening foramen ovale (FO), 7 had a patent ductus arteriosus (DA). Another 18 neonates showed an enlarged RA with a large-in-sized interatrial left-to-right shunting suspected as atrial septal defect.

**Fig. 2:**
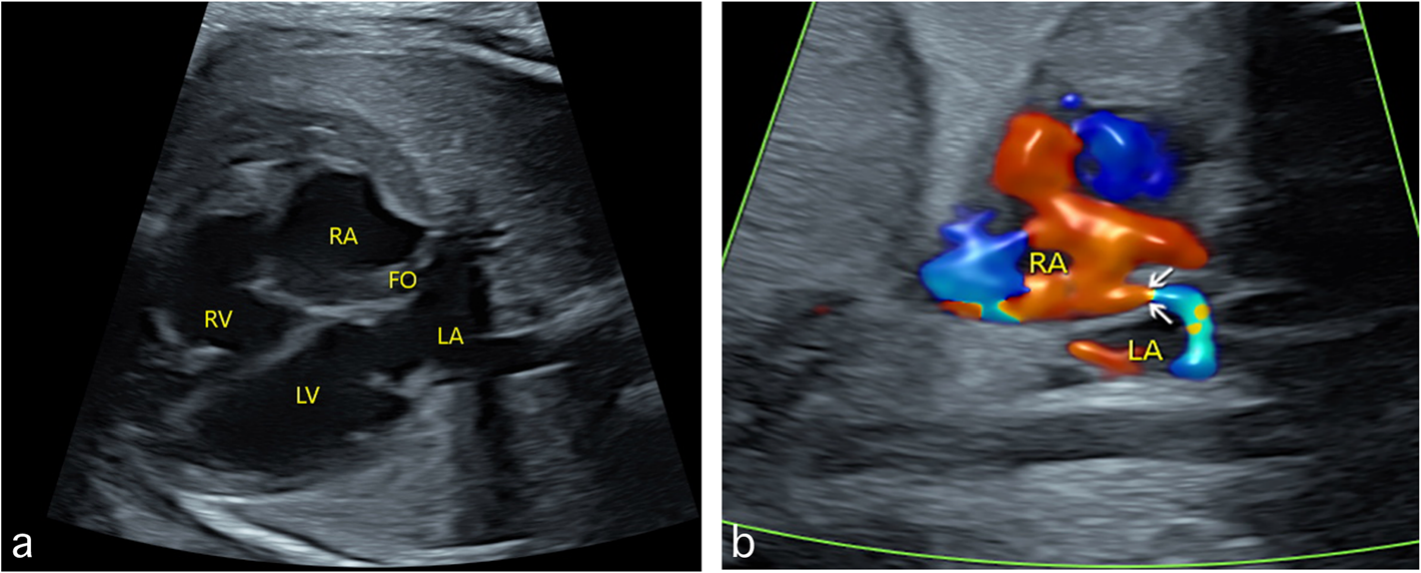
Sonograms showing fetal restrictive foramen ovale. RA dilatation is shown in the four-chamber view (**a**). A sagittal view clearly shows the small gap (indicated by the arrows) between a fixed FO valves and the septum secondum, which is the real entrance of FO to the LA (**b**). FO: foramen ovale; LA: left atrium; LV: left ventricle; RA: right atrium; RV: right ventricle

For fetuses in the last two groups, RA dilatation is a manifestation of abnormal hemodynamics for associated cardiac anomalies, the clinical relevance of which are summarized in Table 1 in the current case series. For these fetuses, the most common reason for RA dilatation is the increasing RA volume, in which the restrictive FO (R-FO) has the highest incidence (*n* = 51). In this malformation, the 4CV can only show an enlarged RA (Fig. 2a). A sagittal view may show the small gap between a fixed FO valves and the septum secundum, which is the real entrance of FO to the LA (Fig. 2b). An additional movie file shows this in more detail (See Additional file 1: Video). For these R-FO fetuses without CHDs in our study, most cases showed a normal RA/LA within the first 2 weeks of life by postnatal echocardiogram. Only 8 neonates with an enlarged RA showed different degrees of TR without other significant cardiac anomalies.

**Table 1:** The clinical relevance of fetuses with right atrium dilatation in our case series

Diagnosis	GW		Associated cardiac anomaly	Outcome		
	19–31	32–36		TOP	IFUD	NNA
Physiological	52	150	TR (101)	0	0	202
Volume overload						
R-FO	3	48	HLHS (9); PV-S (5)^a^; TR (21)	9	0	42
Tricuspid dysplasia	28	15	VSD (3); PE (14); TR (43)	2	0	41
Ebstein’s anomaly	13	3	PV-S (4)^a^; PV-A(3)^b^; VSD(4); TCA(2); PE(2); TR(16)	5	0	11
Tricuspid atresia	9	0	VSD (5); PV-S (5)^a^; HRHS(7); PE(3)	6	0	3
TAPVC	16	3	VSD (5); PLSVC (3)	2	1	16
Galen aneurysm	2	2	Ascites (4); SUA (2)	0	0	4
Pressure overload						
Pulmonary stenosis	32	12	VSD (8); PE (6); PLSVC (2); TR (35)	2	0	42
Pulmonary atresia	13	0	VSD (5); DORV (2); HRHS (4); PE (2); TR (8)	4	0	9
Ductus closure	2	7	VSD (3); RAA (2); PE (3); TR(9)	2	0	7
Ductus contraction	3	7	TR (10)	0	0	10

*DORV* double outlet right ventricle, *GW* gestational weeks, *HLHS* hypoplastic left heart syndrome, *HRHS* hypoplastic right heart syndrome, *IFUD* intrauterine fetal death, *NNA* neonatal alive, *PE* pericardial effusion, *PLSVC* persistent left superior vena cava, *PV-A* pulmonary valve atresia, *PV-S* pulmonary valve stenosis, *RAA* right aortic arch, *R-FO* restrictive foramen ovale, *TAPVC* total anomalous pulmonary venous connection, *TCA* truncus arteriosus, *TOP* termination of pregnancy, *TR* tricuspid regurgitation, *VSD* ventricular septal defect

^a^Cases not repeatedly recorded in the pulmonary valve stenosis group

^b^ Cases not repeatedly recorded in the pulmonary valve atresia group

**Additional file 1: Video.** Color Doppler flow imaging (CDFI) showing the sagittal view of a fetus with restrictive foramen ovale (R-FO) at 32 weeks’ gestation. A sagittal view clearly shows the small gap between a fixed FO valves and the septum secondum, which is the real entrance of FO to the left atrium.

**Fig. 3:**
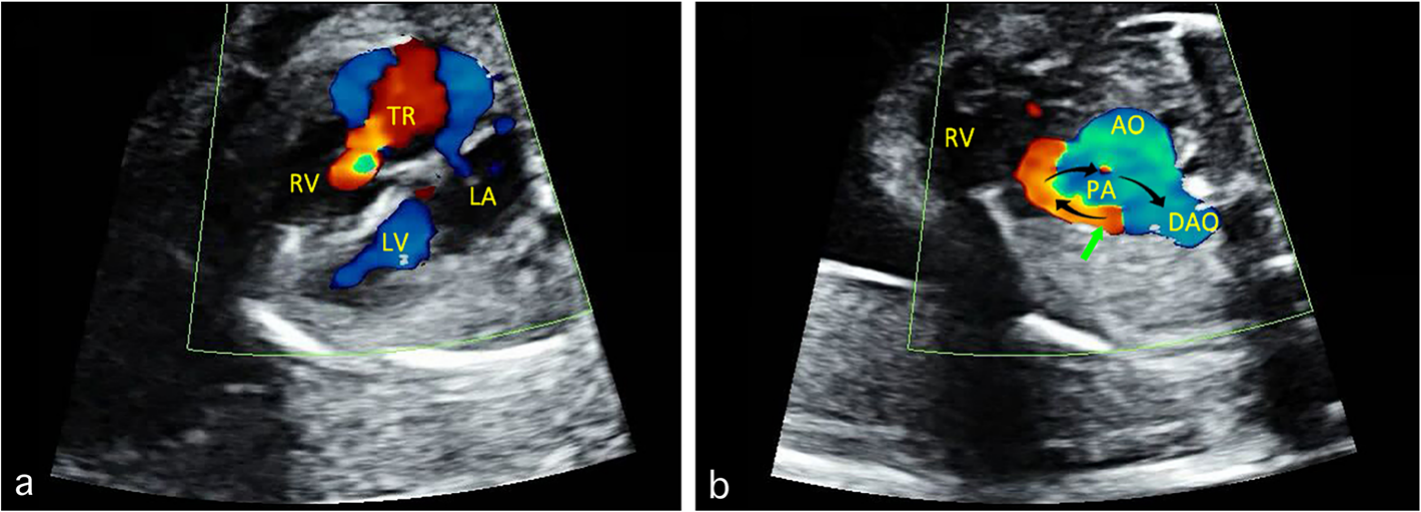
Sonograms showing fetal pulmonary valve atresia. The four-chamber view showed RA dilatation, hypertrophic RV, and severe TR (**a**). The three-vessel trachea view shows that the blood flow direction (indicated by the arrows) in the ductus is from DAO to the PA, instead of the normal PA-DAO direction. In addition, no blood is visualized going through the pulmonary valve (**b**). AO: aorta; DAO: descending aorta; LA: left atrium; LV: left ventricle; PA: pulmonary artery; RA: right atrium; RV: right ventricle; TR: tricuspid regurgitation

Pulmonary abnormalities, including pulmonary valve stenosis and atresia, are common reasons for RA dilatation with an increasing right heart pressure. In a case of pulmonary valve atresia, the 4CV shows an enlarged RA and a hypertrophic right ventricle (RV), together with a severe TR (Fig. 3a). The pulmonary valve is presented as a thick echo dense membrane with no blood visualized going through the pulmonary valve during systole. In addition, the blood flow direction in the ductus is from the descending aorta (DAO) to the pulmonary artery, instead of the normal PA-DAO direction (Fig. 3b). An additional movie file shows this in more detail (See Additional file 2: Video).

### Ratio of right atrium to left atrium

**Fig. 4:**
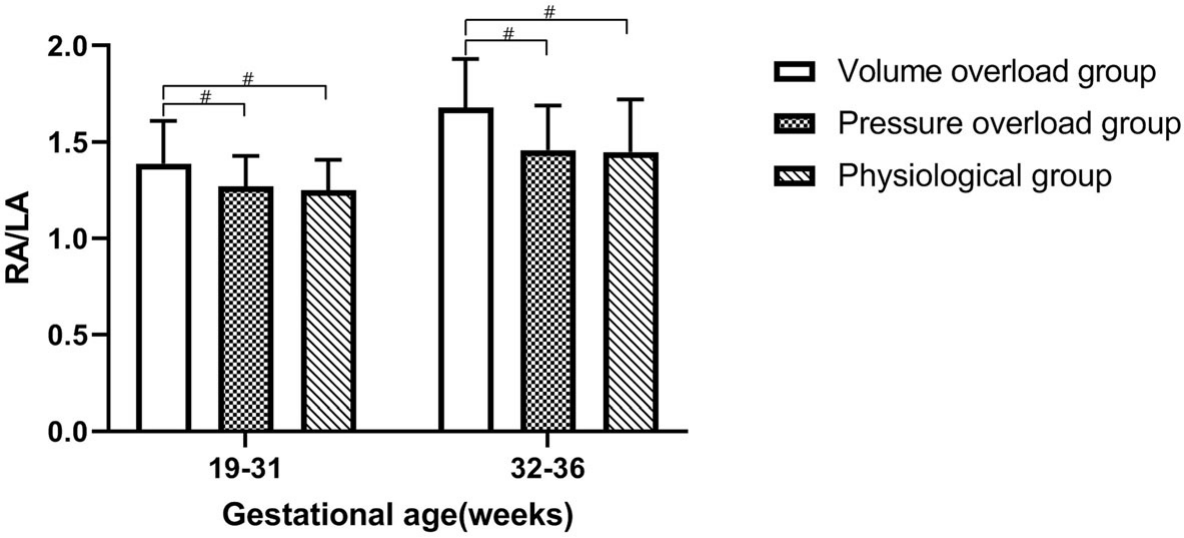
The ratio of fetal right atrium to left atrium (RA/LA) in physiological group, volume overload group, and the pressure overload group according to gestational weeks 19–31, and 32–36. ^#^ indicates *P* < 0.01

In normal circumstances, the size of RA is larger than LA in late pregnancy, especially after 32 gestational weeks (GW). The fetuses were then divided into two subgroups according to gestational age (19-31GW, and 32–36 GW) when assessing the ratio of RA/LA and making comparisons among different groups (Fig. 4). For both subgroups, the RA/LA ratio in the volume overload group is significantly higher than the pressure overload group (both *P* = 0.000) and the physiological group (both *P* = 0.000). However, there is no difference between the pressure overload group and the physiological group for fetuses in both subgroups (*P* = 0.694 for 19–31 GW, and *P* = 0.974 for 32–36 GW, respectively).

### The peak velocity of tricuspid regurgitation in different diseases

As RA dilation is always accompanied by TR and the peak velocity of TR (V_TR_) is easy to obtain during echocardiographic examinations, we made an analysis in fetuses complicated with TR in our case series. The mean V_TR_ for the physiological group, volume overload group and the pressure overload group are (0.88 ± 0.45) m/s, (1.85 ± 0.45) m/s and (3.29 ± 0.58) m/s, respectively. These records showed that V_TR_ in the pressure overload group is significantly higher than both the volume overload group (*P* = 0.000) and the physiological group (*P* = 0.000). In addition, the volume overload group shows a significantly higher V_TR_ than the physiological group (*P* = 0.000).

As the pressure overload group shows a highest value of V_TR_, we further conducted a separate analysis to find the difference between the pulmonary anomalies (including pulmonary valve atresia and stenosis) and the ductal abnormalities (including closure and contraction of DA). The result shows that the latter has a significantly higher value of V_TR_ (3.98 ± 0.41 m/s vs. 3.03 ± 0.38 m/s, *P* = 0.000) than the former.

## Discussion

Measurements of normal cardiac anatomical dimensions, including ventricular diameter, aortic diameter, and so like, have been proposed by many multicenter studies, most of which are positively correlated with gestational age (GA) [12–14]. The normal reference range of fetal heart establishes a quantitative relationship between the cardiac dimensions and the GA, furthermore, it provides an essential reference for clinical evaluation of fetal development. Usually, the atrial size is susceptible to change with the alteration of blood volume [15], which leads to a larger variation especially in the late pregnancy. In comparison, the ratio of RA/LA shows a consistent performance across GA. The fetal four-chamber heart is symmetrical in normal circumstances, with the ratio of RA/LA close to 1: 1 or slightly larger [12]. In the current study, the ratio of RA/LA was then used to evaluate the extent of RA dilatation.

During routine screenings, the examiner is apt to notice an enlarged RA and raises a suspicion of CHD while it remains a challenge to clarify the underlying reasons. We performed an analysis to summarize the anatomical and hemodynamical characteristics of various malformations that may lead to RA dilatation. The relatively large number of cases, together with the prenatal and postnatal data, is a benefit of the research. The reasons for RA dilatation are categorized into physiological enlargement, RA volume overload, and the increasing of pressure in right heart. This classification facilitates the examiners understanding the hemodynamics in different conditions and making an accurate diagnosis, which is another highlight of our study.

Our results suggest that RA volume overload plays an important role in RA dilatation. Due to the lack of muscle fibers in the atrial walls, the RA would expand when the blood volume is increasing, which indicates an intolerance of volume overload for RA. Among various malformations, the restrictive FO is the most common one resulting in RA dilatation. Normally, the FO facilitates oxygen-rich IVC blood to enter the LA, decreasing the volume of blood in the right atrium (right heart) [16]. The FO orifice is easy to visualize at the 4CV. However, the entrance of FO into the left atrium is covered by FO valve, which might stick to the septum secundum to form a restrictive interatrial communication. The entrance of FO to the LA can only be shown at a sagittal plane, which is usually not scanned by screening sonographers.

The increasing of right heart pressure can also lead to RA dilatation, but with a relatively mild extent. However, these types of RA dilatation are always accompanied by severe TR with high velocity [15, 17]. The high-speed TR may produce a pound at the atrial wall to make RA dilatation. In addition, large amount of TR can raise the blood volume in RA, leading to its dilation. However, the interatrial shunt through FO may increase compensably to try to ensure a stable hemodynamics state between the left heart and the right heart, which may decrease the extent of RA dilatation.

When not accompanied with valve diseases, the higher systolic pressure in the right ventricle, the higher V_TR_ would be. It is interesting that the ductal anomaly (closure/contraction) shows a significantly higher V_TR_, when compared with pulmonary valve atresia/stenosis. As the ductus imports more than 90% of the blood flow from the pulmonary artery into the descending aorta, it reduces both the volume and pressure load of the right heart [18]. Obstruction of the ductus is an accurate process which undoubtedly leads to a sharp rise in right ventricular pressure. In comparison, pulmonary valve stenosis/atresia is in a relatively chronic process, which is often accompanied with a ventricular septal defect and/or hypoplastic right ventricle. The interventricular shunt apparently lower down the pressure in the right ventricle, leading to a low-velocity TR. The comparisons made between the DA and PA anomalies in the current research can benefit the sonographers to find the deformity in the case of RA dilatation with increasing pressure in right heart.

## Study limitations

We acknowledge the retrospective nature of our study. The study is also limited that it is a long-period review study, imaging techniques and the examiner’s experience improved with time. The current design was intended to include more cases of various kinds of CHD. In addition, our population had a referral bias, as most of the examinations were done when suspecting of fetal CHDs. However, these limitations, had they been addressed in a multi-center prospective study in a normal screening population, would likely have led to improvements in the completeness of the studies.

## Conclusion

We proposed a strategy to clarify the reasons for RA dilatation by systematically evaluating fetal hemodynamics, which may facilitate the examiner to make an accurate diagnosis of CHD during routine screenings.

## Supplementary information

**Additional file 2: Video.** Color Doppler flow imaging (CDFI) showing the three-vessel trachea (3VT) view of a fetus with pulmonary valve atresia at 24 weeks’ gestation. The 3VT view shows that the blood flow direction (indicated by the arrows) in the ductus is from DAO to the PA, instead of the normal PA-DAO direction. In addition, no blood is visualized going through the pulmonary valve (b). AO: aorta; DAO: descending aorta; PA: pulmonary artery; RV: right ventricle.

## Data Availability

The datasets supporting the conclusions of this article are included within the manuscript (and its additional files). The authors would like to share raw anonymized video data related to the current study, which could only be used for personal study. The demanders may contact baogoubei@hotmail.com

## Abbreviations

RA: Right atrium
RA/LA: The ratio of right atrium to left atrium
4CV: Four-chamber view
TR: Tricuspid regurgitation
V_TR_: Velocity of tricuspid regurgitation
CHD: Congenital heart disease
3VT: Three-vessel trachea
RV: Right ventricle
CDFI: Color Doppler flow imaging
FO: Foramen ovale
R-FO: Restrictive foramen ovale
GA: Gestational age
GW: Gestational week
